# Secretome of human amniotic membrane stem cells promote recovery and testicular functions through modulating *SIRT1/NRF2/TNF-α* pathway in mice testicular torsion: An experimental study

**DOI:** 10.18502/ijrm.v22i10.17670

**Published:** 2024-12-02

**Authors:** Roghayeh Esfehani, Farnaz Khadivi, Jamal Valipour, Maryam Shabani, Mahya Ramesh, Parinaz Javanbakht, Davood Zarini, Sina Mojaverrostami, Masih Hoseini

**Affiliations:** ^1^Department of Anatomy, School of Medicine, Tehran University of Medical Sciences, Tehran, Iran.; ^2^Medical Plants Research Center, Basic Health Sciences Institute, Shahrekord University of Medical Sciences, Shahrekord, Iran.; ^3^Department of Anatomy, School of Medicine, Shahrekord University of Medical Sciences, Shahrekord, Iran.; ^4^Department of Clinical Biochemistry, School of Medicine, Tehran University of Medical Sciences, Tehran, Iran.

**Keywords:** Reperfusion injury, Mesenchymal stem cells, Secretome, Oxidative stress.

## Abstract

**Background:**

Testicular ischemia/reperfusion injury, a significant result of testicular torsion, can lead to the risk of male infertility.

**Objective:**

The current study aimed to evaluate the effect of human amniotic membrane-derived mesenchymal stem cells (hAMSCs) secretome on testicular torsion/detorsion (T/D) in mice.

**Materials and Methods:**

All the experiments were performed in the Anatomy Department of Tehran University of Medical Sciences, Tehran, Iran, during the period of March 2023 to December 2023. 40 male NMRI mice (5–7 wk, 25–30 gr) were randomized into: 1) the sham group: mice received sham operations with no other interventions, 2) T/D group, 3) negative control group; torsion detorsion + intratesticular injection of Dulbecco's Modified Eagle Medium/Nutrient Mixture F-12, and 4) the T/D group + hAMSCs secreted factors. Serum testosterone levels, hematoxylin and eosin staining, and sperm quality parameters were used to evaluate the therapeutic effects of hAMSCs secreted factors on the testicular structure and function. Tissue oxidative stress was measured by determining malondialdehyde, superoxide dismutase, catalase, and glutathione peroxidase-1. Nuclear factor erythroid 2-related factor 2, Kelch-like ECH-associated protein 1, NAD-dependent deacetylase sirtuin-1, tumor necrosis factor-alpha and tumor protein P53 mRNA expressions were assessed in testis via real-time polymerase chain reaction.

**Results:**

The results showed that hAMSCs secreted factors alleviated testicular T/D injury by attenuating oxidative stress, inflammatory response, and apoptosis via modulating the sirtuin-1/ nuclear factor erythroid 2-related factor 2/tumor necrosis factor-alpha signaling pathway.

**Conclusion:**

hAMSCs secreted factors increased antioxidative, anti-inflammatory, and antiapoptotic properties which consequently increased testosterone levels, spermatogenesis, and sperm quality parameters.

## 1. Introduction

One of the common causes of male infertility is testicular torsion (1). According to the previously published study, the prevalence of torsion is 1 in every 4000 men < 25 yr old. This vascular occlusion of the testicle can lead to ischemia and necrosis, and finally lead to loss of spermatogenesis. After the detorsion of the twisted testis, the blood supply to the testicle is suddenly established, which leads to reperfusion of the testicle (2). Therefore, ischemia/reperfusion (I/R) injury is the main pathological and clinical manifestation of testicular torsion and detorsion. Torsion/detorsion (T/D)-induced I/R injury is accompanied by reactive oxygen species (ROS) overproduction, oxidative stress, secretion of interleukin-6 and tumor necrosis factor-alpha (TNF-α), apoptosis and impaired spermatogenesis (3). Oxidative stress refers to an imbalance of the redox system, with free radicals such as ROS accumulating to a level higher than endogenous antioxidative defense (4). It should be mentioned that the presence of small amounts of ROS is necessary to maintain sperm functional processes, including the capacitation period (5).

Oxidative stress in testicular tissue leads to lipid peroxidation of the sperm membrane, DNA damage in sperm, and reduced sperm parameters, ultimately resulting in a reduced fertility rate. Recent studies have shown that the nuclear factor erythroid 2-like 2 (Nrf2)-Kelch-like epichlorohydrin-associated protein 1 (Keap1)-antioxidant response element (ARE) pathway is one of the most important antioxidant signaling pathways (6, 7). Phosphorylated Nrf2 binds to the ARE sequence and transactivates the expression of downstream antioxidant proteins such as heme oxygenase-1, glutathione peroxidase (GPX), and superoxide dismutase (SOD) (8). The Sirtuin family regulates cell death, cell survival, and oxidative stress response through histone deacetylation (9). In the sirtuin-1 (Sirt1)/Nrf2/TNF-α pathway, the *Sirt1* gene is the upstream factor and enhances the expression of the Nrf2 (10).

Mesenchymal stem cells (MSCs) have different therapeutic effects against various diseases through antioxidative and immunomodulatory effects. MSCs can be isolated from different tissues, including bone marrow, adipose tissue, umbilical cord, amniotic membrane, and fetal sources (11). Human amniotic membrane-derived mesenchymal stem cells (hAMSCs) are a promising source of MSCs. They can be easily obtained without ethical concerns because these fetal membranes are usually considered hospital waste (12).

In addition, hAMSCs are considered to be a promising source for stem cell therapy due to their low immunogenicity, non-tumorigenicity, multi-differentiation potential, and lack of ethical problems associated with embryonic stem cells (13). The human amniotic membrane is a cheap biological resource because its extracellular matrix contains different cytokines, including fibroblast growth factor, hepatocyte growth factor, epidermal growth factor, vascular endothelial growth factor, etc. (14). MSCs can potentiate the antioxidant defense of the testicular tissue by reducing ROS production and inhibiting oxidative stress (15). Transplantation of MSCs can be considered a practical solution to these problems (16). The MSCs-derived cell-free secretome was shown to have many properties of MSCs (17).

Hence, in the present study, the therapeutic effects of hAMSCs on testicular T/D were assessed using antioxidative markers, sperm parameters, serum testosterone level and the Sirt1/Nrf2/TNF-α signaling pathway.

## 2. Materials and Methods

### Experimental animal design

In this experimental study, 40 male NMRI mice (5–7 wk, 25–30 gr), were purchased from Pasture Institute (Karaj, Iran). Animals were housed at a standard temperature of 24 ± 3 C and maintained under a stable 12-hr light/dark cycle. All the experiments were performed in the Anatomy Department of Tehran University of Medical Sciences, Tehran, Iran, from March 2023 to December 2023. The animals were fed a standard pellet diet and water ad libitum. Mice received surgical T/D at the age of 6–8 wk, following a 10-day acclimatization period. Based on the histological and molecular analyses and in accordance with our previous studies, the number of samples in each group were 10 mice (18). All surgical procedures were done under aseptic and sterile conditions. First, animals were anesthetized through intraperitoneal injection of ketamine/xylazine (10:1 ratio). Then, the surgical site was shaved and sterilized. After that a midline incision was made, and the left spermatic cord was rotated 720 in a counterclockwise direction.

Spermatic cord torsion was maintained by suturing the tunica albuginea of the testis to the scrotal wall. After 2 hr of torsion, the fixing suture was removed, the left spermatic cord was untwisted 720 in a clockwise direction and the testis was placed in its normal anatomical position in the scrotum. Animals were randomly allocated into 4 groups:

Sham group: animals underwent midline scrotal incision, and the left testis was only picked up and then returned to its normal position. T/D group: animals received testicular T/D surgery. Negative control group: animals underwent left testicular torsion surgery, and 10 μ L Dulbecco's Modified Eagle Medium (DMEM)/F-12 was injected locally into the left testis 1.5 hr after torsion surgery or 30 min before detorsion.

T/D group + hAMSCs-secreted factors (treatment group): animals received left testicular torsion surgery, and 10 μ L hAMSCs secreted factors were injected locally into the left testis 1.5 hr after torsion surgery or 30 min before detorsion (18). After surgical intervention, animals were returned to their cages under auto-regulated thermal light to maintain their body temperature at 37 C.

### Preparation of secreted factors from hAMSCs culture

The term placenta from healthy women was obtained immediately after delivery, and the amniotic membrane was separated from the chorion. Afterward, the amnion was cut on small pieces and decontaminated in phosphate-buffered saline (PBS) (Gibco, USA) containing 100 μ g/ml streptomycin and 100 U/ml penicillin. DMEM medium containing 0.5 mg/ml collagenase type IV (Gibco, USA), 0.25% trypsin (Gibco, USA), and 1% pen/strep were used for incubation of fragments for 15 min at 37 C. The obtained hAMSC were centrifuged, and then filtered with 100 μ m cell strainers, and washed with PBS.

Harvested cells were cultured in 25 cm^2^ flasks with DMEM/F-12 medium supplemented with 1% penicillin-streptomycin (Gibco) 10% fetal bovine serum (Gibco, Paisley, UK). Twice a week, the cultured cells were passaged. To obtain hAMSCs secreted factors, we used cells from 3–5 generations. After that the MSCs at 70–80% confluence in 25 cm^2^ culture flasks were washed 3 times with PBS to wipe out residual FBS and cell debris. hAMSCs for every flask were incubated in DMEM/F-12 medium without adding FBS for another 48 hr. The supernatant was then collected, extant cells and debris were picked up by 5 min centrifugation with 1500 RPM and then supernatant was filtered by a 0.22 μ m filter. Finally, the supernatant was freeze-dried 15 times and finally, hAMSCs secreted factors were preserved at -80 C for subsequent analysis (18).

### hAMSC characterization by flow cytometry analysis

We used flow cytometry to characterize the purity of hAMSCs using the following markers: CD45 (negative marker) and CD73 (positive marker). A total of 1 × 10^6^ hAMSCs were used for flow cytometry. 100 μ L cell suspensions of hAMSCs were centrifuged. The supernatant was removed, and the cell pellet was preserved and then fixed for 10 min with a 4% formaldehyde solution (Merck KGaA, Darmstadt, Germany). Then, the obtained hAMSCs were incubated with phycoerythrin (PE)-conjugated primary polyclonal antibody or fluorescent isothiocyanate against hAMSC of positive marker and negative marker for 40 min at room temperature (all antibodies were bought from BD Biosciences, USA). Flow cytometry was analyzed on stained cells and unstained control cells presumed as negative control (19).

### Measurement of the left testicular weight

Animals were sacrificed after 35 days of T/D surgery. Testicular tissue specimens were exposed from the scrotum, and the average weights of testes were recorded in experimental groups. The tissues were then homogenized in PBS and stored at -80 C for further investigations.

### Determination of sperm parameters

To acquire mature spermatozoa from the epididymis, the caudal portion or tail of the epididymis was cut into small pieces and placed in a prewarmed petri dish containing 2 mL of PBS. Tissue samples in the petri dish were torn using a scalpel blade to facilitate sperm diffusion within the solution. Tissue samples were mechanically incised using a scalpel blade to facilitate sperm diffusion within the solution. Then Petri dishes containing sperm suspension were incubated at 37 C in the incubator supplied with 5% CO_2_ for 20 min (20).

Sperm motility was assessed according to the World Health Organization 2010 recommendation. Single 10 µl drops of prepared sperm suspension were placed on a preheated slide. The motility of 200 spermatozoa in 10 microscopic fields was estimated at x400 magnification using a light microscope (Nikon, Tokyo, Japan). Finally, the percentages of progressive motile spermatozoa, nonprogressive motile spermatozoa, and immotile spermatozoa were reported.

10 μ l of sperm suspension was placed on each slide and smears were prepared. The slides were air-dried at room temperature and then stained using the diff-quik staining protocol. According to this protocol, the dried smear was first immersed in triarylmethane dye and methanol as a fixative 5 times and allowed to air dry completely. After that the slide was immersed in solution I (xanthene dye, sodium azide, pH buffer) 3 times, each time for 10 sec. Then, the slide was immersed in solution II (thiazine dye, pH buffer) 5 times, each time for 10 sec.

Excess stain was swiftly washed with sterile water, and then the slide was dried at room temperature. Eventually, after mounting the slide was covered with a coverslip. At least 200 spermatozoa were analyzed in each assessment utilizing light microscopy at a magnification of x1000. Viable cells remained colorless while dead spermatozoa stained pink or dark pink. Sperm morphology was also evaluated with this diff-quik staining method.

The procedure described in previous studies with slight modification was applied for the evaluation of total sperm count per sample (21). The total sperm count was expressed as 10^6^ sperm/ml.

10 μ l of sperm suspension was taken on each slide and a smear was prepared. The slides were air-dried at room temperature and fixed with 70% ethanol for 30 min. Samples were stained according to Papanicolaou's kit method (Fra fan slamt paydar, Iran). At least 200 spermatozoa were evaluated under a light microscope at a x100 magnification. They were examined in terms of normal and abnormal morphology.

### Serum testosterone analysis

The mice were anesthetized with ketamine/xylazine mixture (10:1 ratio, ip injection) and the blood sample was collected from the heart with a 21-gauge needle from the left ventricle and then centrifuged to obtain serum. The serum was collected and stored at -20 C for testosterone assay.

The total testosterone concentration was measured from the serum by using an enzyme-linked immunosorbent assay (ELISA) kit (MONOKIT Testosterone ELISA Kit, M24L1L2, OBL, Iran) according to the manufacturer's instructions. The validation of the human ELISA kit in animal experiments (rats and mice) has been described previously (22). Briefly, 10 μ L of standard, control, and sample were added to each well of the ELISA plate. Then 50 μ L of the enzyme conjugate and 50 μ L of the testosterone antiserum were added to all of them.

After 1 hr incubation with gentle continual shaking (500–700 rpm) at room temperature, each well was washed with washing buffer. Then, 100 μ L tetramethyl benzidine substrate was added to each well and incubated for 15 min in darkness. After adding 50 μ L of stop solution (0.2 M sulfuric acid) to each well, the absorbance was measured using a microplate reader (OD: 450 nm).

### Measurements of malondialdehyde (MDA) and antioxidative enzymes levels

The level of MDA content has long been utilized as a useful biomarker for oxidative stress and lipid peroxidation. The level of MDA in testes was used to measure lipid peroxidation.

The thiobarbituric acid (Nalondi TM Lipid Peroxidation Assay Kit-MDA) method was used to detect MDA levels and lipid oxidation in the testes caused by torsion-detorsion injury. The samples were homogenized with lysis buffer and butylated hydroxytoluene. The absorption of supernatants was measured at 550 nm after centrifugation. MDA concentrations were determined in samples based on the standard curve and results were presented as nmol/mg protein.

SOD enzymatic activity level was determined based on its ability to inhibit pyrogallol autoxidation which extremely depends on the activity of superoxide (Nasdox^TM^- Superoxide Dismutase Assay Kit-Nonenzymatic). This method is according to the capability of SOD for inhibition of pyrogallol autoxidation and the absorbance change was detected at 405 nm. The results were expressed as U/mg protein.

Catalase (CAT) activity was evaluated according to the manufacturer's instructions (Nactaz^TM^- Catalase Activity Assay Kit-CAT). This test was based on the utilization of H_2_O_2_ at 550 nm. CAT activity was reported according to the standard curve and results were indicated as nmol/mg protein.

GPX activity was assessed according to the commercially available kit method (Nagpix^TM^- Glutathione Peroxidase Assay Kit). The oxidation of NADPH to NADP + coincides with a reduction in absorbance at 340 nm (A340). This is a spectrophotometric analysis for detecting GPX enzyme activity then the results were expressed as nmol/mg protein.

### Real-time polymerase chain reaction (PCR)

The relative expression of oxidative stress-related, inflammation-related, and apoptosis-related genes was evaluated by quantitative real-time PCR. Total RNA was extracted from fresh left testicular tissue samples using the GeneAll Ribx protocol (GeneAll, South Korea). The purity and concentration of the extracted RNA were assessed using a nanodrop spectrophotometer. cDNA was synthesized using the GeneAll cDNA synthesis kit (GeneAll, South Korea). Quantitative real-time PCR (qRT-PCR) was done utilizing an applied biosystems real-time PCR system (USA) and FIREPol EvaGreen qPCR master mix (Solis BioDyne, Estonia). Following amplification conditions were used: initial activation at 95 C for 15 min, followed by 40 cycles of denaturation at 95 C for 30 sec, and annealing/extension at 60 C for 60 sec. The β -*actin* gene was used as a housekeeping gene. The relative expression (fold change) in the mRNA expression level was calculated using the equation ΔΔ CT and normalized to the control group expression. Table I lists the specific primers used in this experiment.

**Table 1:** Primer sequences used for real-time PCR analysis

**Gene name**	**Sequences**	**Melting temperature**	**Product size**
*** α-Actin***	F: 5 ' -ATGGAGCCACCGATCCAC-3 ' R: 5 ' -CATCCGTAAAGACCTCTATGCCAAC-3 '	59.0 61.4	171
***Sirt1***	F: 5 ' -GTCTTGTCCTCTAGTTCCTGTG-3 ' R: 5 ' -GCCTCTCCGTATCATCTTCC-3 '	57.8 57.2	133
***Nrf2***	F: 5 ' -GGAGAGGATGCTGCTGAAAG-3 ' R: 5 ' -CCTCGCTGGAAAAAGAAGTG-3 '	58.3 57.0	80
***Keap-1***	F: 5 ' -CTTAGGGTGGATGCCTTCGAT-3 ' R: 5 ' -CTGCCCAATTCATGGCTCACA-3 '	59.8 61.2	125
***TNF-α ***	F: 5 ' -TGCTCTGTGAAGGGAATGGG-3 ' R: 5 ' -ACCCTGAGCCATAATCCCCT-3 '	59.6 60.0	142
***p53***	F: 5 ' -AACCGCCGACCTATCCTTAC-3 ' R: 5 ' -CACAAACACGAACCTCAAAGC-3 '	59.2 58.5	88
PCR: Polymerase chain reaction, β -*Actin*: Beta actin, *Sirt1*: NAD-dependent deacetylase sirtuin-1, *Nrf2*: Nuclear factor erythroid 2-related factor 2, *Keap-1*: Kelch-like ECH-associated protein 1, *TNF- α *: Tumor necrosis factor-alpha, *p53*: Tumor protein P53, F: Forward, R: Reverse			

### Ethical Considerations

This study was approved by the Institutional Animal Care and Use Committee of the Tehran University of Medical Sciences, Tehran, Iran, for the care and working with laboratory animals (Code: IR.TUMS.AEC.1401.122).

### Statistical Analysis

All statistical analyses were performed using the SPSS software (Version 22.0, IBM Corp., Armonk, New York) and all graphs were generated with Prism 7.0 (GraphPad software). All data are reported as mean ± standard deviation (SD). Shapiro-Wilk tests were used to assess the normality of the data. For multiple comparisons, one-way analysis of variance (ANOVA) followed by post hoc Tukey's test was used to test statistical significance. A p-value < 0.05 was considered as statistically significant.

## 3. Results

### Histopathological analysis

**Figure 1:**
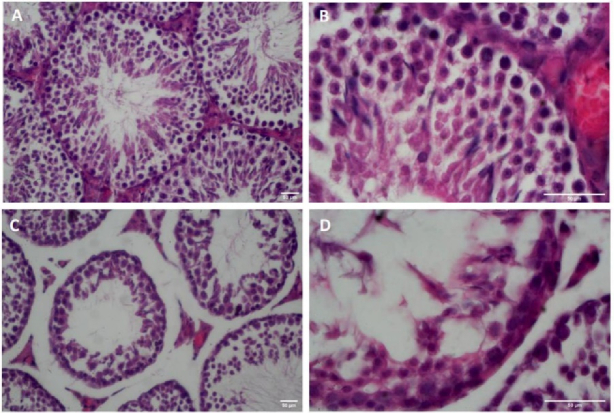
Histomorphological evaluations of the left testicular tissues with H&E-staining. A and B) Sham group, C and D) T/D group. A, C) x400 magnification. B, D) x1000 magnification. Scale bar: 50 µm.

Light microscopic examination of testis tissue sections from the Sham-operated group showed the typical histomorphological architecture of seminiferous tubules with regular normal germinal epithelial cells. The spermatogenic cells are organized into distinct layers in the cross-section of seminiferous tubules. The germ cells are supported by somatic Sertoli cells within the tubules and Leydig cells can be observed between the seminiferous tubules. The cross-section of seminiferous tubules in the T/D group displayed abnormal histomorphology with disorganization and severe sloughing of the germinal epithelium. As seen in figure 1, the number of germ cells and diameter of the tubules were also decreased in the T/D group.

### Characterization of hAMSCs by flow cytometry 

**Figure 2:**
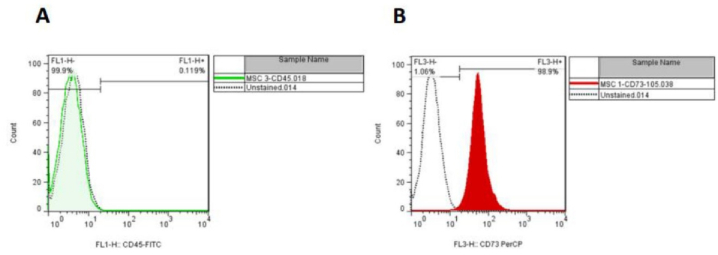
Characterization of hAMSCs. Flow cytometry histograms show that A) hAMSC were negative for CD45, while B) Positive for CD73.

The result of flow cytometry analysis displayed the expression of CD73 (positive marker). Low levels of CD45 (negative marker) have been reported in cultured cells (Figure 2).

### Testicular weight evaluation in experimental groups

Left testicular weight decreased significantly in the T/D group compared to the Sham group (p < 0.001). Although left testicular tissue weight significantly increased in the T/D + hAMSC-secreted factors group compared to the T/D group (p = 0.03), no statistically significant difference was observed compared to the Sham group (Table II).

### Evaluation of serum testosterone levels in experimental groups

Serum testosterone levels in the T/D group significantly decreased compared to the sham group (p < 0.001). Serum testosterone levels significantly increased after intratesticular injection of hAMSC-secreted factors compared to the T/D group (p < 0.001). No significant difference was observed between the Sham and T/D + hAMSC-secreted factors groups (Table II).

### Evaluation of oxidative stress and antioxidant enzyme activities in experimental groups

**Figure 3:**
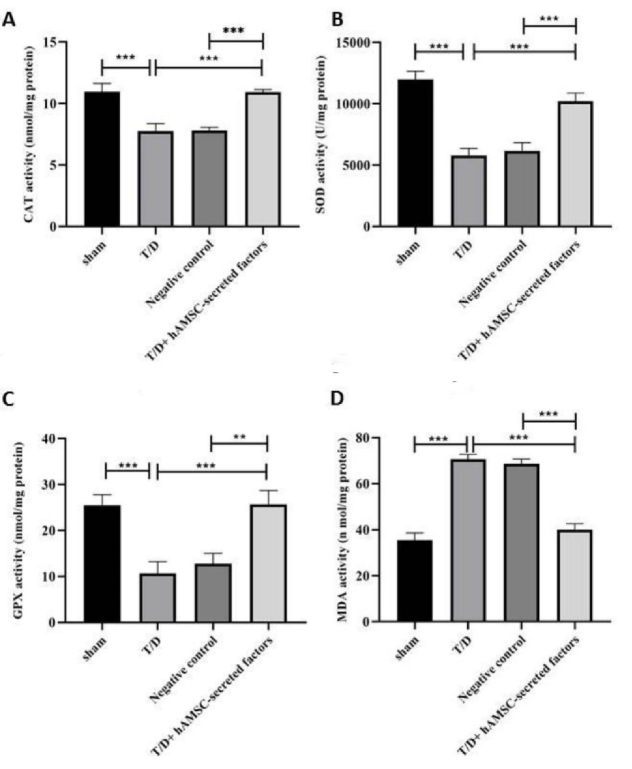
Antioxidant and oxidative stress-related enzymes were detected by ELISA assay. A) CAT, B) SOD, C) GPX, D) MDA. Values were presented as Means ± SD. ***P < 0.001, **P < 0.01 used for significant differences between groups. hAMSCs: Human amniotic membrane-derived mesenchymal stem cells, T/D: Testicular torsion/detorsion. CAT: Catalase, SOD: Superoxide dismutase, GPX: Glutathione peroxidase, MDA: Malondialdehyde.

MDA levels were significantly increased (p < 0.001) and CAT, GPX, and SOD activities were significantly reduced (p < 0.001, p < 0.001, p < 0.001, respectively) in the T/D group compared to the Sham group. Our results in the T/D + hAMSC-secreted factors group showed a significant decline in MDA levels (p < 0.001) and a dramatic increase in CAT, GPX, and SOD activities (p < 0.001) compared to the T/D group. No statistically significant difference was observed in MDA levels or antioxidant enzyme activities between the T/D + hAMSC-secreted factors and sham groups (Figure 3).

### Evaluation of sperm parameters (motility, viability, count, and morphology) in experimental groups

Evaluation of sperm parameters showed that epididymal sperm count significantly decreased in the T/D group compared to the sham group (p < 0.001). Although sperm count in the T/D + hAMSC-secreted factors group significantly increased as compared to the T/D group (p < 0.001), a significant difference was also observed compared to the sham group (p < 0.001).

A considerable reduction in sperm motility was reported in the T/D group compared to the sham group (p < 0.001). Sperm motility significantly increased after injection of hAMSC secretome compared to the T/D group (p < 0.001), but not as much as the sham group (p < 0.001).

Sperm viability was significantly decreased in the T/D group compared to the sham group (p < 0.001). Sperm viability in the T/D + hAMSC-secreted factors group dramatically increased compared to the T/D group (p < 0.001), and the sperm viability results showed statistically significant differences between the sham and T/D + hAMSC-secreted factors groups (p < 0.001).

**Figure 4:**
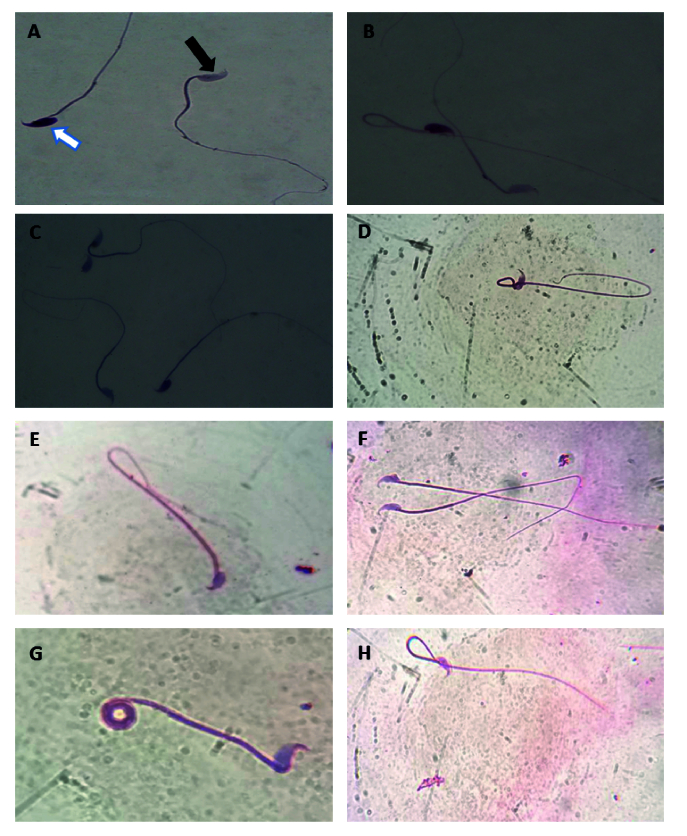
Sperm viability was evaluated by Diff-Quik staining. A) Unstained sperm (alive) with a colorless head (black arrow), stained sperm (dead) with a pink color head (white arrow), B and C) Sperm abnormality was evaluated by Diff-Quik. D, E, F, G, and H) Sperm abnormalities. E) Hairpin, F) Broken, G) Coiled, H) Hairpin loop, was evaluated by the Papanicolaou method. Magnification: x1000.

Our results demonstrated that normal sperm morphology dramatically reduced after T/D surgery compared to the sham group (p < 0.001). The hAMSC secretome noticeably improved sperm morphology compared to the T/D group (p < 0.001); however, a significant difference was still observed compared to the sham group (p < 0.001) (Table II, Figure 4).

### Evaluation of relative mRNA expression levels of inflammatory mediators, oxidative stress genes, and apoptosis genes in experimental groups

**Figure 5:**
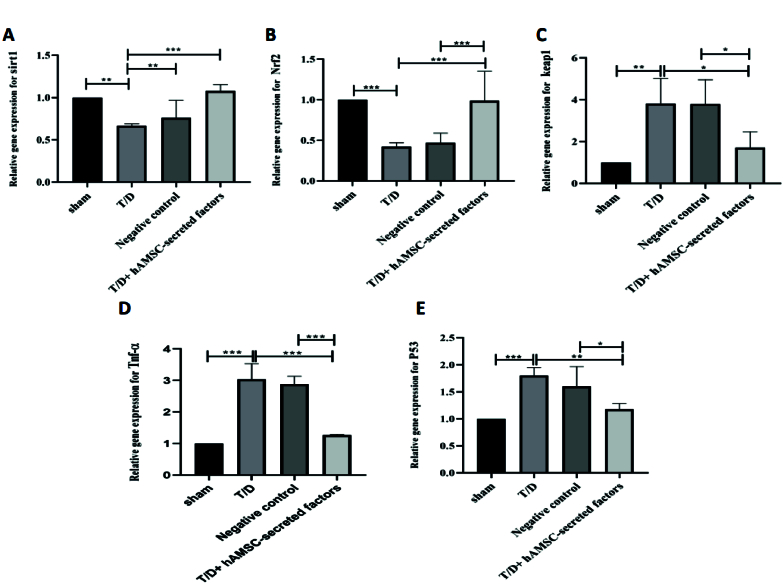
qRT-PCR assay showed mRNA levels calculated by ΔΔ CT formula for A) *Sirt1*, B) *Nrf2*, C) *Keap1*, D) *Tnf- α *, and E) *P53* genes. Values were shown as Means ± SD. 3 replications were performed on 5 samples in each group. ***P < 0.001, **P < 0.01, *P < 0.05 used for significant differences between groups. hAMSCs: Human amniotic membrane-derived mesenchymal stem cells, T/D: Testicular torsion/detorsion. PCR: Polymerase chain reaction, β -*actin*: Beta-actin, *Sirt1*: NAD-dependent deacetylase sirtuin-1, *Nrf2*: Nuclear factor erythroid 2-related factor 2, *Keap-1*: Kelch-like ECH-associated protein 1, *Tnf- α *: Tumor necrosis factor-alpha, *p53*: Tumor protein P53.

Real-time PCR analysis was used to determine the relative expression of *TNF- α *, an inflammatory factor, and *P53*, a major indicator of apoptosis. The relative expression levels of *TNF- α* and *P53* were significantly increased in the T/D group compared to the sham group (p < 0.001, p < 0.001, respectively) (Figure 5). Our results showed that intratesticular injection of hAMSC-secreted factors considerably reduced the relative expression of *TNF- α* and *P53* compared to the T/D group (p < 0.001, p = 0.001), but not as much as in the sham group (Figure 5).

In addition to inflammatory and apoptosis-associated genes, the relative expression of *Nrf2*, *Keap1*, and *Sirt1* were also evaluated. Our findings revealed no statistically significant differences in the relative expression of *Sirt1* between the groups (Figure 3). The relative expression of the *Nrf2* gene in mouse testicular tissue after T/D surgery was remarkably downregulated compared to the sham group (p < 0.001) (Figure 5). As shown in figure 3, the relative expression of *Nrf2* was significantly increased after injection of hAMSC-secreted factors compared to the T/D group (p < 0.001). In line with the results of *Nrf2*, the relative expression of *Keap1* was significantly upregulated in the T/D group compared to the sham-operated group (p < 0.001). However, the relative expression of this gene significantly declined after intratesticular injection of hAMSC-secreted factors (p < 0.001) (Figure 5).

**Table 2:** Sperm parameters in different experimental groups

**Groups**	**Sham**	**T/D**	**Negative control**	**T/D + hAMSCs secreted factors**
**Sperm count (×10^6^)**	29.07 ± 0.30 ${\;}^{\text{ab}}$ (p < 0.001)	6.7 ± 0.26^a^ (p < 0.001)	6.633 ± 0.25^a^ (p < 0.001)	24.67 ± 0.65^b^ (p < 0.001)
**Progressive motility (%)**	20.68 ± 0.01 ${\;}^{\text{ab}}$ (p < 0.001)	9 ± 0.2^a^ (p < 0.001)	9 ± 0.1^a^ (p < 0.001)	16.7 ± 0.06^b^ (p < 0.001)
**Nonprogressive motility (%)**	38.94 ± 0.36 ${\;}^{\text{ab}}$ (p < 0.001)	15.62 ± 0.54^a^ (p < 0.001)	15.64 ± 0.55^a^ (p < 0.001)	38.71 ± 0.15^b^ (p < 0.001)
**Immotile sperm (%)**	40.38 ± 0.01^b^ (p < 0.001)	75.38 ± 0.01^a^ (p < 0.001)	75.36 ± 0.01^a^ (p < 0.001)	44.59 ± 0.01^b^ (p < 0.001)
**Total motility (%)**	59/62 ± 0.01 ${\;}^{\text{ab}}$ (p < 0.001)	24.62 ± 0.02^a^ (p < 0.001)	24.64 ± 0.01^a^ (p < 0.001)	55.41 ± 0.01^b^ (p < 0.001)
**Viability rate (%)**	66 ± 1 ${\;}^{\text{ab}}$ (p < 0.001)	26.40 ± 0.7^a^ (p < 0.001)	26.93 ± 1.7^a^ (p < 0.001)	56.77 ± 1^b^ (p < 0.001)
**Normal morphology (%)**	88.39 ± 1.07 ${\;}^{\text{ab}}$ (p < 0.001)	41.67 ± 1.5^a^ (p < 0.001)	41.59 ± 1.15^a^ (p < 0.001)	66.74 ± 1^b^ (p < 0.001)
**Abnormal head morphology (%)**	3.86 ± 0.01 ${\;}^{\text{ab}}$ (p < 0.001)	10.83 ± 0.01^a^ (p < 0.001)	10.80 ± 0.015^a^ (p < 0.001)	3.96 ± 0.01^b^ (p < 0.001)
**Abnormal tail morphology (%)**	7.93 ± 0.01 ${\;}^{\text{ab}}$ (p < 0.001)	47.50 ± 0.01^a^ (p < 0.001)	47.51 ± 0.02^a^ (p < 0.001)	29.30 ± 0.1^b^ (p < 0.001)
**Left testis weight (mg)**	106.3 ± 5.5^b1^ (p < 0.001)	64 ± 4^a1^ (p < 0.001)	63.67 ± 3.38^a1^ (p < 0.05)	92 ± 4.34^b1^ (p < 0.001)
**Serum testosterone (pg/ml)**	0.2633 ± 0.01^b1^ (p < 0.001)	0.0341 ± 0.004^a1^ (p < 0.001)	0.044 ± 0.008^a1^ (p < 0.001)	0.233 ± 0.058^b1^ (p < 0.001)
Data presented as mean ± SD. One-way ANOVA was used for multiple comparisons, and statistical significance between experimental groups was determined by post hoc Tukey's test. Statistical significance was defined as p < 0.05. b: p < 0.0001, b1: p < 0.001 used for significant differences as compared to T/D group. a: p < 0.0001, a1: p < 0.001 used for significant difference as compared to T/D + hAMSCs secreted factors group. hAMSCs: Human amniotic membrane-derived mesenchymal stem cells, T/D: Testicular torsion/detorsion				

## 4. Discussion

This study assessed the ameliorative effect of hAMSCs secreted factors on testicular I/R injury following testicular T/D. Serum testosterone levels increased after injection of hAMSC-secreted factors, which is in accordance with the results of a previous study that showed that hAMSCs were used for the treatment of male infertility in animal models (23).

The formation of ROS after I/R injury is the main pathological mechanism of testicular T/D. Lipid peroxidation of cell membranes, DNA damage, and apoptosis in testicular germ cells were reported as consequences of elevated ROS levels (24). MDA levels in testis tissue were measured as an indicator of oxidative damage. The results of the present experiment showed that MDA levels and antioxidant enzyme activities of SOD, GPX, and CAT significantly reduced following T/D surgery, similar to the findings of previous studies (25). Antioxidant defense systems, such as SOD, GPX, and CAT, are capable of shielding organisms from ROS.

Studies have shown that a common regulatory factor NRF2, regulates the production of antioxidant enzymes. NRF2 regulates transcriptions of downstream genes with antioxidant response elements, such as CAT, SOD, and GPX (26, 27). Increased ROS generation consumes several antioxidant enzymes, so excessive oxidative stress releases Nrf2 in testicular T/D injury. The relative mRNA expression of the *Nrf2* gene decreased in the T/D group compared to the sham group. In addition to the oxidant-antioxidant imbalance, there is also an inflammatory response after I/R injury. Overproduction of pro-inflammatory cytokines such as TNF-α, and interleukin-1 β , expression of vascular adhesion molecules, and the testicular influx of neutrophils are the main reasons for testicular damage after T/D intervention (28).

Significant susceptibility of spermatozoa to oxidative stress, related to the high content of polyunsaturated fatty acids in the plasma membrane. We investigated the effect of T/D on epididymal sperm parameters and found a significant decline in sperm count, motility, morphology, and viability. Our findings were consistent with the previous study that reported a severe decrease in sperm motility and viability after testicular T/D injury in a mouse model (29). A significant relationship was observed between *NRF2* mRNA levels and specific functional parameters of sperm, such as motility, abnormal morphology, and DNA fragmentation (30).

We observed a significant increase in the relative mRNA expression of the *P53* gene after testicular T/D as an index of apoptosis. Similarly, we have seen a significant decrease in the number of spermatogenic cells in sections of seminiferous tubules after following the testicular T/D surgery (18). In addition, increased testicular apoptosis in rat testes after testicular T/D was confirmed in another study (31).

*Sirt1*, a histone deacetylase can adjust cellular stress response and longevity (32). In T/D-induced I/R injury in juvenile rats, *Sirt11* expression significantly decreased in the testis (10). In the *Keap1-Nrf2-ARE* signaling pathway, *Sirt1* is a key regulator factor of *Nrf2*, and it finally upregulates the expression of *NRF2* downstream genes, such as SOD (6, 7).

In the Keap1-Nrf2 complex, Keap1 acts as a negative regulatory protein targeting Nrf2 from ubiquitin-dependent proteasomal degradation under physiological conditions. *Sirt1* deacetylates *Nrf2*, increasing its stability and enhancing the expression of antioxidant genes (33). Upon oxidative stress, *Nrf2* detaches from *Keap1*, replaces to the nucleus, and binds to antioxidant response elements. This binding trans activates the expression of downstream antioxidant proteins, including SOD, CAT, and GPX (8).

The secretion of stem cells contains various bioactive and immunomodulatory factors, and its application may be an option for regenerating damaged tissue (34). Despite the many benefits of MSCs transplantation against various disorders in testes, some disadvantages have been reported, such as tumor formation, unwanted differentiation, and blockage of the nutritional microvasculature (16). However, by using the secretome of MSCs instead of the MSCs themselves, these problems can be solved. By bypassing the challenges of using stem cells directly, MSCs secretions offer a promising way to deliver their healing benefits.

The secretome of MSCs eliminates the risk of blood clots, cell rejection, excessive cell growth, and tumor formation (11, 35). Moreover, secretions are easier to manipulate, store, and package than MSCs directly. Epidermal growth factor, a component of the hAMSC secretome, reduces oxidative stress by activating the *Nrf2* pathway (17). We demonstrated that intratesticular injection of hAMSC-derived secretome significantly restored testicular weight. Similarly, hAMSC transplantation to busulfan-induced azoospermic rats restored testicular size and weight (23). The results of this study revealed that the hAMSC secretome improved sperm viability, motility, total number, and morphology, and reduced the proportion of morphologically abnormal sperm. Consistent with our findings, AD-MSCs conditioned medium was shown to improve sperm quality in the presence of H_2_2O_2_-induced oxidative stress (17). Previously, hAMSC secretome improved total sperm number, increased the proportion of rapidly moving sperm, and significantly inhibited the proportion of immotile sperm in cisplatin-induced testicular dysfunction (23). Recently, another study reported that adipose tissue-derived MSCs conditioned medium increased sperm number and motility, as well as the total number of testicular cells, in cisplatin-induced testicular damage in mice (19). The results of the current study showed that the hAMSC secretome activates the *Keap1-Nrf2-ARE* signaling pathway, increasing the levels of antioxidant enzymes SOD, GPX, and CAT and decreasing the level of MDA. Confirming our findings, previously it has been reported that conditioned medium of human placental MSCs stimulated the *Nrf-2* signaling pathway and defend cells against oxidative damage (36). Increased *Nrf2* expression and prevention of its depletion after treatment inhibited oxidative stress and inflammation.

Our findings showed that the relative mRNA expression of the *TNF- α* and *P53* genes significantly decreased after intratesticular injection of hAMSCs-derived secreted factors. Previously, administration of human umbilical cord multipotent mesenchymal stromal cells for treating acute I/R injury in rats diminished the inflammatory response. Besides, administration of hUC-MSCs reduced the ROS level, decreased germ cell apoptosis and enhanced spermatogenesis (37).

## 5. Conclusion 

In the present study, intratesticular injection of hAMSCs secreted factor following testicular T/D improved antioxidative, anti-inflammatory, and antiapoptotic properties, which consequently increases testosterone levels, spermatogenesis, and sperm quality parameters. We found that these beneficial effects were associated with modulating the Sirt1/Nrf2/TNF-α signaling pathway. These findings can be attributed to the paracrine secretions of hAMSCs suggesting that hAMSCs may provide an effective therapeutic strategy for the treatment of male infertility. The current study had some limitations such as hAMSCs secretome characterization and proteomic analysis were not performed, local injection of secretome can damage seminiferous tubules and destroy the testicular niche, and clinical translation is difficult due to the difference in germline cells of different species in many aspects. It would be suggested to use different doses of secretome in future studies, also, it would be valuable to evaluate the effect of various doses in rescuing ischemia-reperfusion injury. We expect our study to pave the way for new research and therapeutic implications of hAMSCs secretome in improving spermatogenesis and fertility in male reproductive disorders.

##  Data Availability

Data supporting the findings of this study are available upon reasonable request from the corresponding author.

##  Author Contributions

R. Esfehani: Methodology, writing- original draft, F. Khadivi: Conceptualization, writing- review and editing, validation. J. Valipour: Writing- review and editing, validation, methodology. M. Shabani: Project administration, methodology, M. Ramesh: Methodology, writing- review and editing, P. Javanbakht: Methodology, writing- review and editing, D. Zarini: Project administration, methodology, writing- review and editing, S. Mojaverrostami: Writing- original draft, validation, supervision, project administration, methodology, conceptualization, M. Hosseini: Writing- review and editing, validation.

##  Conflict of Interest

The authors declare that there is no conflict of interest.
